# Ceilings of treatment: a qualitative study in the emergency department

**DOI:** 10.1186/s12873-019-0225-6

**Published:** 2019-01-17

**Authors:** Nathan Walzl, Jessica Jameson, John Kinsella, David J. Lowe

**Affiliations:** 1 School of Medicine, University of Glasgow, Wolfson Medical School Building, University Avenue, Glasgow, G128QQ UK; 2 Emergency Department, Monklands Hospital, Airdrie, UK; 3 Academic Unit of Anaesthesia, Pain and Critical Care Medicine, School of Medicine, University of Glasgow, Glasgow, UK; 4 Emergency Department, Queen Elizabeth University Hospital, Glasgow, UK

**Keywords:** Ceiling of treatment, Decision making, Emergency medicine, Qualitative research

## Abstract

**Background:**

Decision-making concerning the limitation of potentially life-prolonging treatments is often challenging, particularly in the Emergency Department (ED). Current literature in this area of Emergency Medicine is limited and heterogeneous. We seek to determine the factors that influence ceiling of treatment institution in the ED.

**Methods:**

We conducted a phenomenological qualitative study employing semi-structured interviews. Emergency Medicine Consultants were recruited via a sample of convenience from 5 hospitals in the West of Scotland. Data saturation was achieved after 15 interviews. Interviews were recorded, anonymised, transcribed, coded, and an iterative thematic analysis was carried out.

**Results:**

A model was created to illustrate the identified themes. Patient wishes are central to decision-making. Acute clinical factors and patient-specific factors lay the foundations of ceiling of treatment decisions. This is heavily contextualised by family input, collateral information, anticipated outcome, and whether the patient is accepted for higher care. This decision-making process flows through a ‘filter’ of cultural and environmental factors. The overarching nature of patient benefit was found to be of key importance, framing all aspects of ceiling of treatment institution. Ultimately, all ceiling of treatment decisions result in one of three common patient pathways: full escalation, limited escalation, and maintenance of current care with the option of palliative care initiation.

**Conclusions:**

We present a conceptual model composed of 10 major thematic factors that influence Consultant ceiling of treatment decision-making in the ED. Clinicians should be cognizant of influential factors and associated biases when making these important and challenging decisions.

**Electronic supplementary material:**

The online version of this article (10.1186/s12873-019-0225-6) contains supplementary material, which is available to authorized users.

## Background

Physicians in the Emergency Department (ED) frequently encounter decisions concerning the institution of an appropriate level of treatment for patients presenting with critical illness [1]. As most developed countries are faced with an ageing and increasingly multimorbid population [2, 3], the need to develop a rational approach to end-of-life care in the ED is becoming more important and has been highlighted as a research priority in Emergency Medicine [4].

The term ceiling of treatment has yet to be conclusively defined in the literature and there has been limited exploration of the issue within the context of Emergency Medicine [5]. Here, a ceiling of treatment is considered to be the predetermined highest level of intervention deemed appropriate by a medical team, aligning with patient and family wishes, values and beliefs. These crucial early decisions aim to improve the quality of care for patients in whom they are deemed appropriate. Judiciously instituted ceilings of treatment help improve patient and family experience of the dying process through the recognition and allowance of natural death, whilst avoiding the excessive allocation of scarce resources to provide futile life sustaining treatments [6, 7].

In the UK there is a legal duty of care that requires patient views to be incorporated into clinical decision-making to ensure treatments are aimed to the overall benefit of the patient. This includes a patient’s ultimate right to accept or refuse treatment. Should they lack capacity, decisions made on the patient’s behalf must also be the least restrictive of their future choices, and the healthcare team must take account of any views or preferences expressed by the patient, including advance decisions, and must seek any consent required from legal proxies [8].

Decision-making concerning the limitation of potentially life-prolonging treatments is frequently challenging. It is perceived as particularly complex in the ED as physicians rarely know the patient, crucial data is often lacking, patients frequently lack capacity at the time of decision-making, and the prevalence of Advance Directives remains low [1, 8–10]. A further challenge is the narrow time frame within which ceiling of treatment decisions are often made, despite patients commonly presenting with multiple complications and several complex conditions [1, 11].

Notwithstanding considerable inter-physician and inter-departmental variation in ED ceiling of treatment decision-making, the evidence base is limited [12–14]. A previously conducted short-form literature review summarises the current literature using a range of methodologies [5]. Factors identified through observational studies can be broadly categorised into patient and disease factors [1, 15–19]. Qualitative literature identified suggests that implementation of ceilings of treatment is further affected by physician, timing and unit-related factors; legal and moral considerations have also been identified as important [20, 21]. How the above factors are combined and their respective weighting and influence on the decision to institute ceilings of treatment is variable and remains poorly understood [22].

Identifying the factors and biases that underpin ceiling of treatment decision-making in the ED may provide useful guidance for physicians making these challenging decisions and inform educational programmes. A qualitative approach is needed to expand the limited literature and validate the transferability of previous findings to current clinical practice in Emergency Medicine. This study aims to determine the factors that influence the institution of ceilings of treatment in the ED.

## Methods

### Study design

A phenomenological approach was used in this qualitative study. The goal of phenomenological research is to describe the world as experienced by individuals, in order to discover the common meanings underlying variations of a given phenomenon [23]. Phenomenology complements the objectives of this study in developing a deeper understanding of ceiling of treatment institution through the understanding of several individual’s common or shared experiences. Ultimately, we aimed to develop a theoretical model of ceiling of treatment institution in the ED, placing emphasis on factors that drive decision-making.

Interviews took place in five university-affiliated hospitals in the West of Scotland, representing a mix of tertiary referral centres and district general hospitals. Fifteen interviews were conducted from January to March 2017.

### Samples

Participants were University of Glasgow affiliated Emergency Medicine Consultants. Consultants are the highest-grade doctors and senior decision makers in the National Health Service (NHS). Participants were identified via convenience sampling by D.J.L., and subsequently recruited via individual email. Participant number was determined by data saturation (when no new themes emerge during the iterative coding process), which was achieved after 15 interviews. Participant demographics are shown in Table 1.

**Table 1:** Demographic characteristics of study participants

		Total, No.
Hospital	QEUH	6
	GRI	3
	Monklands	3
	RAH	2
	Hairmyres	1
Sex	Male	8
	Female	7
		Years
Experience as Consultant [median (interquartile range)]		5 (4–7)

*QEUH* Queen Elizabeth University Hospital, *GRI* Glasgow Royal Infirmary, *RAH* Royal Alexandria Hospital

### Data collection methods

Data was collected through semi-structured interviews and recorded using an electronic data-recording device. Interviews were directed by a pre-defined schedule (Additional file 1) based on ‘current understanding’, ‘challenges’ and ‘improvements’ concerning ceilings of treatment. The semi-structured interview schedule was tested and improved during initial interviews. Before the interviews were carried out, discussions and interview training took place. D.J.L. attended an initial interview conducted by N.W., who conducted all subsequent interviews. D.J.L. reviewed audio and transcribed data throughout the data collection process to verify quality and consistency. N.W. drafted the manuscript and all authors contributed substantially to its revision.

### Data analysis

All interviews were transcribed verbatim by a professional transcription service. Thematic data analysis was carried out in an iterative process throughout the study. Recorded audio was repeatedly listened to, verifying the integrity of transcribed data.

Initially, N.W., D.J.L. and J.J. read and reread three transcripts individually, coding data into index themes. This ‘inclusive’ process of constant comparison was repeated iteratively until all factors from each interview were inductively organised into analytical categories [24]. The process formed a seminal coding framework, which N.W. used to code remaining interviews, integrating emerging codes into the coding framework. Qualitative analysis software (NVivo; QRS International Pty Ltd.; version 11, 2015) was used throughout the data analysis process.

### Trustworthiness

Trustworthiness and methodological rigour was established in keeping with Lincoln and Guba’s evaluative criteria [25]. Credibility was demonstrated through regular peer debriefing, member checking via responder validation, and availability of interview transcripts on request. Transferability was established through a rich description of the context in which this study was conducted and data collection to saturation. Dependability was evidenced by stepwise replication ensuring coding agreement from researchers and detailed audit trail of the data collection process. Finally, confirmability was established through reflexive journals kept throughout the data collection and analysis process, frequent investigator meetings, and confirmability audit ensuring that interpretation of data clusters were reasonable and meaningful.

### Ethics

Approval was obtained from the local ethics committee. Participants were provided with participant information sheets and completed written informed consent documents. Interview transcripts were anonymised.

## Results

### Thematic analysis

**Fig. 1:**
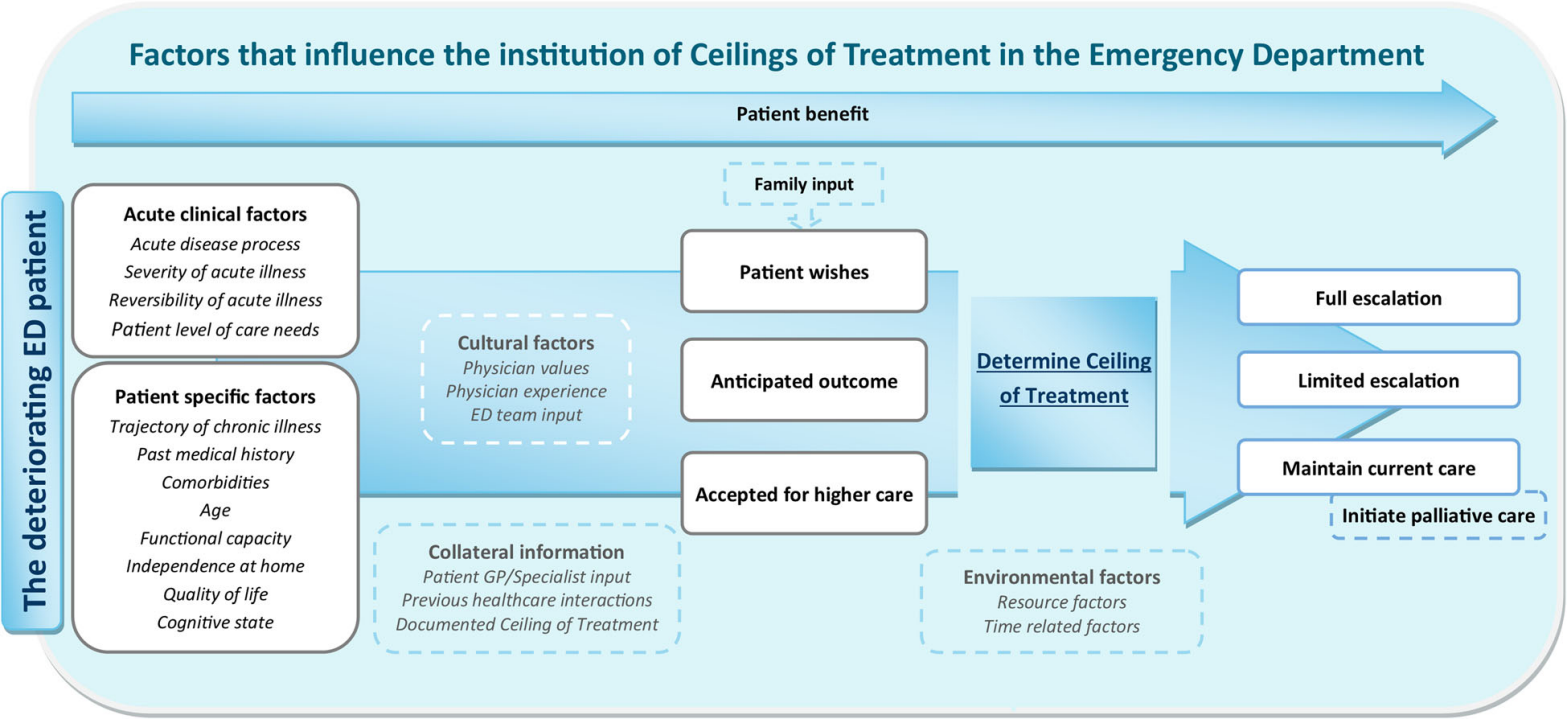
Model illustrating factors influencing ED Consultant ceiling of treatment decision-making as identified through thematic analysis

Ten overarching major themes and twenty supporting themes were identified through thematic analysis as influential in ED Consultant ceiling of treatment decision-making. A conceptual model was created to illustrate these findings (Fig. 1). Major and supporting themes are illustrated with quotes in Table 2 and described in greater detail below.

**Table 2:** Major themes and supporting thematic factors identified, with illustrative quotes

Major Themes	Supporting Themes	Illustrative Quotes
Patient benefit		*“…I work out what would be the maximal humane or tolerable treatment…”*
Acute clinical factors	Acute disease process	*“… if their condition was such that it was just for palliation. So if someone had, say, ischaemic bowel, I might decide that given their age and issues that they might not be for surgical intervention…”*
	Severity of acute illness	*“…that ceiling of care process is probably there for every patient I see… it just comes to the fore when a patient is particularly sick…”*
	Reversibility of acute illness	*“…something that was entirely reversible, I wouldn’t even ask the family’s opinion prior to getting them admitted to intensive care.”*
	Patient level of care needs	*“I tend to start at the top and decide whether or not somebody who is sick is for intensive care, and I work my way down until I get to a ceiling…”*
Patient specific factors	Trajectory of chronic illness	*“If the patient has terminal cancer I may decide… if they’re coming in with respiratory failure from a chest infection… I’m not going to proceed to intubation.”*
	Past medical history	*“Given their past medical history… you’re coming to the conclusion that the patient is never going to intensive care…”*
	Comorbidities	*“…this presenting complaint already has a high mortality attached, but in this patient’s case, who has a lot of comorbidities, it’s going to be even higher…”*
	Age	*“I think with younger people, so under 75, we tend to be more aggressive as a whole, but if I’ve got a well-functioning 86-year-old then it doesn’t play a role…”* *“I think age comes into it… it probably shouldn’t but it definitely does.”*
	Functional capacity	*“If you’ve got a very unwell, chronically sick, debilitated 20-something year old, your ceiling of care might be lower than that of a much older patient who is physically very fit…”*
	Independence at home	*“… somebody who is in a nursing home… are very much delineated markers for what level of care would be placed.”*
	Quality of life	*“Do they still enjoy something? If that’s watching the tennis or just going out into the garden… if they’re not getting any of that, what are we trying to achieve?”*
Anticipated outcome		*“There was no dispute in his diagnosis, the boy was going to die. It’s where, who with, and under what circumstances he was going to pass away.”*
Accepted for higher care		*“…you can talk about the medical things you can do, but the neurosurgeon made the decision for you.”* *“…a decision that’s already made by the intensive care doctors, if they’ve had one admission… It will occasionally be documented that the patient will not be accepted for intensive care treatment again.”* *“I’ve worked across England, and ICUs are quite individual things… some ICUs just have very different levels for admission…”*
Patient wishes		*“We’re not going to put them on NIV because the patient has decided they never want that again…”*
Family input		*“…then the ceiling of care is determined by the next of kin… we would potentially disagree with it… but you’ve got to involve the families.”* *“we obviously use them [family], but it’s usually a clinical or medical decision… sometimes their expectations are far from realistic…”*
Collateral information	Patient’s GP or specialist input	*“Depending on who’s around and what time of day it is, I might phone the GP… a clinician that’s been involved in their care…”*
	Previous healthcare interactions	*“You’ll find out their end-of-life decision making process because…you’ll read up what’s happened in the clinics…”*
	Documented CoT decision	*“…all I needed to do was follow the plan that had been previously agreed…”*
Cultural factors	Physician values	*“I would want my child no matter what… that was the right thing to do…”* *“There are some people that would continue to resuscitate…and just don’t want patients to die. With the best will in the world they will decide to keep going… and I’m not one of them.”*
	Physician experience	*“You’ve made that decision before…it’s an easier decision to make…”*
	ED team input	*“I make these decisions with the team looking after them. If a nurse has been looking after one patient for six hours… they’ll probably know more about… the whole package that goes with them than me…”* *“…working in a team that doesn’t function well… might lead them to change their decision-making process…”*
Environmental factors	Resource factors	*“…there are only a finite number of resources... ICU is no different, they’ve got a finite number of beds. And that may rightly or wrongly play a part.”*
	Time related factors	*“It’s very time consuming for us, practically, so if we say we’re going to palliate someone at home… most of the time it’s impossible because a lot of our work is out of hours, and palliative care and primary care is 9–5, Monday to Friday.”* *“I think if it had been four in the morning she’d probably have gone down the same route I had, but when you get… a standby call at 7:40… you’re switched off and concentrating on not falling asleep…”*

*ED* Emergency Department, *NIV* non-invasive ventilation, *GP* general practitioner, *CoT* ceiling of treatment, *ICU* intensive care unit

#### Patient benefit

The continuous framing of ceiling of treatment decisions around clinician-perceived patient benefit was a ubiquitous finding, and respondents almost universally stated early in the interviews that doing the best thing for the patient formed the basis of all subsequent decisions. This overarching theme was frequently expressed through discussions concerning ‘good death’, ‘harm versus benefit’ and ‘futility’ of interventions. Patient benefit was principally felt to be an inferred judgement in situations where input from the patient, surrogate decision makers, or family was unattainable.

#### Acute clinical factors

The *acute disease process* was discussed by many respondents as part of an assessment of the patient’s current illness. Uncertainty surrounding the underlying acute disease process was perceived as making ceiling of treatment decisions challenging.

Most respondents considered *severity of acute illness* based on clinical, physiological and biochemical variables to be an important factor. The ‘very unwell patient’ was often described as a ‘trigger’ of consideration or initiation of ceilings of treatment.

*Reversibility of acute illness* was considered by most participants to be important in decision-making, with two common scenarios highlighted: the first being lack of reversibility due to the nature and severity of acute presentation (such as devastating brain injury), and the second being lack of response to initial treatment. Decision-making in situations where the patient’s condition was thought to be reversible was described as straightforward with escalation to critical care considered the likely appropriate level of intervention.

Finally, the patient’s *level of care needs* were considered in terms of surgical interventions, medical therapy and nursing care. Many respondents shared the default ‘stance’ of initially considering all patients to be for full escalation, establishing the appropriateness of full active treatment, and working ‘down’ the treatment levels in a step-wise manner.

#### Patient specific factors

The process of gathering information about a patient’s *past medical history* was described as crucial by some respondents before an appropriate level of treatment could be identified. Most participants felt that presentations resulting from a *chronic disease process* heavily influence ceiling of treatment decisions. End-stage COPD and advanced malignancy were frequent examples.

Many participants discussed the impact of *comorbidity* on ceiling of treatment decisions, which some respondents felt stemmed from an increase in mortality associated with multi-morbidity.

*Age* was discussed by the majority of respondents. Many felt that age should not impact decision-making, basing their decisions instead on the patient’s functional status, citing frequent discrepancy between ‘biological’ and ‘chronological’ age. Nonetheless, some respondents acknowledged that age impacted their decision-making, especially for young patients. Others discussed age as a rough indicator of physical and cognitive reserve.

Some Consultants felt that patient pre-morbid *functional capacity* was an important factor, as further deterioration in level of function was seen as a likely outcome of severe illness. ‘Frailty’ and ‘fitness’, as well as physiological concepts of cardiopulmonary reserve were discussed. Additionally, some respondents discussed the importance of patient *cognitive state* as a factor to be considered.

Patient *independence at home* was described by some respondents as influential. This was often expressed through ‘activities of daily living’ and patient mobility, as well as the environment in which the patient is normally cared for. *Quality of life* was frequently considered important, often involving a subjective assessment of the patient’s pre-morbid quality of life as well as a prediction of the extent to which it would be impacted by any interventions being considered. Several respondents perceived the determination of quality of life to be challenging due to the vast variation in what is considered an acceptable quality of life between individuals.

#### Anticipated outcome

All participants stated that anticipated patient outcome plays a critical role in decision-making. It was generally felt that ceilings of treatment are frequently instituted when death is likely to be imminent. Participants commonly voiced aversion to admitting patients who ultimately “are never going to come off the ventilator” or recover from ICU. In such situations, where death is perceived to be highly likely, ceilings of treatment are instituted in the interest of patient benefit.

#### Accepted for higher care

All respondents discussed that the level of treatment escalation is determined through a process of joint decision-making with receiving specialties. Participants felt that ceilings of treatment are sometimes determined by whether receiving specialties such as intensive care or surgery accept a patient for escalation of treatment on acute presentation. Previously documented decisions specifying that surgery or re-admission to intensive care would not be appropriate for a patient in the context of their illness were frequently cited by Consultants as influential. Some respondents stated that ICUs can differ in terms of admission criteria and thus felt that decision-making is swayed by local ICU admission culture.

#### Patient wishes

All respondents felt that patient wishes are both important and central to decision-making. Consultants considered patients with capacity (and their legal proxies) to be the final authority concerning decisions in their own care, even when this was at odds with the course of action perceived to be most beneficial. Many participants acknowledged that the majority of patients for whom a ceiling of treatment may be appropriate in the ED lack the ability to express their wishes. This was described as a significant clinical challenge.

#### Family input

Most respondents felt that family wishes and opinions play an important role in decision-making. The importance placed on family input was often perceived as context dependent, with added emphasis when information regarding the patient’s own wishes is lacking, and reduced emphasis in the context of families with unrealistic expectations.

#### Collateral information

Some respondents described a process of information seeking from the *patient’s GP or specialists* such as their oncologist due to their familiarity with the patient’s illness, baseline status and wishes. This approach was often perceived to be limited to normal working hours.

*Previous healthcare interactions* were described as influential by some respondents. Medical notes, discussions from clinics, past admissions to ICU or ward-based care were seen as important sources of information, especially when managing patients with pre-existing conditions.

Many participants stated that, when present, *documented ceiling of treatment decisions* have a significant impact on decision-making. Do Not Attempt CPR forms, Advance Directives and Anticipatory Care Plans were mentioned, but their rarity was frequently remarked upon. Respondents perceived decisions involving previously discussed and agreed upon ceilings of treatment to be more straightforward.

#### Cultural factors

Most participants felt that *physician values*, beliefs and personality traits influence ceiling of treatment institution. Some perceived this to be a source of variability in decision-making. A few participants referred to what level of intervention they would want instituted for themselves or a family member as justification for ceiling of treatment decisions made for patients.

*Physician experience* was discussed by around half the respondents. Many felt that experience making ceiling of treatment decisions was important when judging what level of care fits the patient’s best interests. Participants often referred to junior doctors, who were perceived as more likely to proceed with full treatment than senior decision makers. Two respondents also felt that personal specialist skill or experience such as intensive care background also affect decision-making.

Some participants noted that *input from ED team members*, including nursing staff and Consultant colleagues, can play a role in decision-making. Participants felt that the limitation of treatment is a team decision and found benefit from consulting ED colleagues on challenging cases. One respondent felt that a poorly functioning team could alter decision-making, and another expressed the view that decision-making may be shaped to conform to departmental attitudes.

#### Environmental factors

Respondents felt that *resource availability* should not play a role in determining ceilings of treatment. Nonetheless, many expressed the opinion that factors such as intensive care occupancy, staff availability and department demand can influence the level of treatment instituted for patients.

*Time related factors* were discussed by several respondents as impacting decision-making in two main ways: firstly, the logistical and practical implications of instituting a ceiling such as home-based palliative care in an out-of-hours, busy ED were described as monumental. Secondly, some participants felt that decision-making can be contextually affected by timing factors, such as the end of a nightshift.

Finally, a conceptual model was created to illustrate the major and supporting themes identified (Fig. 1).

## Discussion

Describing the factors that affect ceiling of treatment decision-making in the ED is important, as timely and informed decision-making facilitates effective and early delivery of patient-centred care. This qualitative study elucidated several factors that influence the institution of ceilings of treatment by senior decision makers in the ED*.* The factors identified are complex, heavily context-dependent and their weighting ambiguous. Nonetheless, it should be noted that decisions were ultimately universally viewed through the lens of patient benefit, reflecting an emphasis on beneficence as a guiding ethical principle underpinning ceiling of treatment institution [26].

Our results suggest that patient wishes are central to ceiling of treatment decision-making, albeit frequently opaque. Acute clinical factors and patient specific factors lay the foundations of ceiling of treatment decisions. Such case-specific information is heavily influenced by family input, collateral information, the anticipated outcome and whether the patient is accepted for higher care. This process flows through a ‘filter’ of cultural and environmental factors. The overarching nature of patient benefit was found to be of key importance, framing all aspects of ceiling of treatment institution. Ultimately, decisions around determining an appropriate ceiling of treatment for a given patient result in of one of three common pathways: full escalation, limited escalation, and maintenance of current care with the option of palliative care initiation.

It was observed that many Consultants shared the common approach of initially considering all patients to be for full treatment by default. A decision-making process aiming to establish an appropriate ceiling of treatment for the patient is then triggered, working ‘down’ the treatment levels in a step-wise manner continuously influenced by the factors previously mentioned.

To our knowledge, this is the first investigation to describe the factors that affect ED ceiling of treatment decision-making in the UK, and the first to look exclusively at senior decision makers. Findings are highly consistent with clinical and patient-specific factors previously elucidated by observational studies and show strong similarity to qualitative data previously published in France and Australia [1, 9, 14–19].

Interestingly, some identified factors have not been previously discussed in the ED literature in relation to ceiling of treatment decision-making, including resource factors and the impact of ED team input [5]. The finding that ICUs can differ in terms of admission criteria has been established in the intensive care literature; however the impact resulting from variation in local ICU admission culture on ED ceiling of treatment decision-making had not previously been elucidated [27].

The finding that patient wishes are central to decision-making is in line with the ethical principle of respect for autonomy and current legislation [8, 26]. However, respondents generally felt that patient wishes can only be discerned in a minority of cases for whom a ceiling of treatment is considered in the ED. This highlights the importance of adequate advance planning to support decision-making for patients presenting to the ED with a critical event.

This study has some significant limitations. The data was acquired from a relatively small sample of Consultants in the West of Scotland, limiting generalisability to other units and to healthcare systems with different staffing or organisational models. Specifically, the UK model for healthcare provision is significantly different than that of privatised systems. It should be noted that the median respondent experience as a Consultant was 5 years and in part reflects the recent expansion in Consultant numbers in Scotland. We accept that different views may have been elicited from a sample of more experienced practitioners or with wider geographical sampling, and that as a result of the sample of convenience used during participant recruitment the sample may not have been fully representative of each unit. Finally, whilst there may be value in expanding the generalisability of findings to different grades, specialties, and other healthcare professionals, this was not within the scope of this study.

## Conclusions

This paper enhances current understanding of ED ceiling of treatment decision-making, highlighting key factors and important biases physicians should be cognizant of when making these challenging decisions. Perhaps our most important finding is the creation of a novel conceptual model that highlights 10 major factors which influence ceiling of treatment decisions made by Consultants, the most senior decision makers in the NHS. The weighting of the respective identified factors remains unclear, and their influence on the decision to institute a ceiling of treatment is variable and heavily context dependent. There is scope for further research on this topic to validate and further extend the generalisability of our findings.

## Additional file

Additional file 1: Interview Schedule. Interview schedule used to direct all semi-structured interviews conducted during this study. (PDF 132 kb)

## Abbreviations

AAA: Abdominal Aortic Aneurysm
COPD: Chronic Obstructive Pulmonary Disease
CoT: Ceiling of Treatment
CPR: Cardiopulmonary Resuscitation
ED: Emergency Department
GP: General Practitioner
GRI: Glasgow Royal Infirmary
ICU: Intensive Care Unit
NHS: National Health Service
NIV: Non-Invasive Ventilation
QEUH: Queen Elizabeth University Hospital
RAH: Royal Alexandria Hospital
