# Supplementary material for: Ceilings of treatment: a qualitative study in the emergency department
Source: BMC Emerg Med. 2019 Jan 17;19:9. doi: 10.1186/s12873-019-0225-6 (PMC6335704; doi:10.1186/s12873-019-0225-6)
Supplement: Supplementary file 1 — Interview Schedule. Interview schedule used to direct all semi-structured interviews conducted during this study. (PDF 132 kb) [file 12873_2019_225_MOESM1_ESM.pdf]

SUPPLEMENTARY FILE 1

Interview schedule

<u>Questionnaire</u>

- What year did you graduate from University? ___________
- What university did you graduate from? ___________
- Where did you do your training? ___________
- How many years have you been a consultant? ___________

<u>Interview Schedule</u>

Verify the patient has signed the consent form. Use questions to generate detailed responses

1. What do you understand a ‘ceiling of care’ to be?

2. Can you tell me a little bit about the process of instituting a ceiling of care?

3. Can you tell me about a time when instituting a ceiling of care has particularly challenged you?
    a. *Can you tell me about a time when instituting a ceiling of care was easy?*

4. When you found yourself in a difficult decision making situation, how did you make it easier?

5. Have you experienced any contradictions between ceilings of treatment and the patient’s best interests?

6. What do you think would help improve decision making?
